# Health coaching to improve self-management and quality of life for low income patients with chronic obstructive pulmonary disease (COPD): protocol for a randomized controlled trial

**DOI:** 10.1186/s12890-017-0433-3

**Published:** 2017-06-09

**Authors:** Beatrice Huang, Rachel Willard-Grace, Denise De Vore, Jessica Wolf, Chris Chirinos, Stephanie Tsao, Danielle Hessler, George Su, David H. Thom

**Affiliations:** 1 Department of Family and Community Medicine, University of California San Francisco, San Francisco, CA USA; 2 San Francisco Department of Public Health, San Francisco, CA USA; 3 Department of Medicine: Pulmonology, Critical Care, Allergy and Sleep Medicine Program, University of California San Francisco, San Francisco, CA USA

**Keywords:** Health coaching, Chronic obstructive pulmonary disease, Quality of life

## Abstract

**Background:**

Chronic obstructive pulmonary disease (COPD) severely hinders quality of life for those affected and is costly to the health care system. Care gaps in areas such as pharmacotherapy, inhaler technique, and knowledge of disease are prevalent, particularly for vulnerable populations served by community clinics. Non-professionally licensed health coaches have been shown to be an effective and cost-efficient solution in bridging care gaps and facilitating self-management for patients with other chronic diseases, but no research to date has explored their efficacy in improving care for people living with COPD.

**Method:**

This is multi-site, single blinded, randomized controlled trial evaluates the efficacy of health coaches to facilitate patient self-management of disease and improve quality of life for patients with moderate to severe COPD. Spirometry, survey, and an exercise capacity test are conducted at baseline and at 9 months. A short survey is administered by phone at 3 and 6 months post-enrollment. The nine month health coaching intervention focuses on enhancing disease understanding and symptom awareness, improving use of inhalers; making personalized plans to increase physical activity, smoking cessation, or otherwise improve disease management; and facilitating care coordination.

**Discussion:**

The results of this study will provide evidence regarding the efficacy and feasibility of health coaching to improve self-management and quality of life for urban underserved patients with moderate to severe COPD.

**Trial registration:**

ClinicalTrials.gov identifier NCT02234284. Registered 12 August 2014.

## Background

Chronic obstructive pulmonary disease (COPD) is the 3rd leading cause of death in the United States [1] with estimated healthcare costs of $36 billion dollars in 2010 [2]. More than 15 million Americans are diagnosed with COPD, and with 50% of people living with COPD thought to be undiagnosed, the number of people affected is likely to be higher [1].

At least half of all COPD patients do not receive recommended pharmacologic therapies [3–5], and many never see a pulmonologist. Even with correct pharmacologic therapies, inappropriate use of medications still poses a barrier to COPD care. In a study of inhaler use, over 65% of patients demonstrated poor technique with at least one inhaler device [6]. The burden of caring for COPD in addition to many other chronic diseases typically falls on primary care providers, who may not have the time nor training to appropriately address this issue [4, 7].

Because of its burden to the health care system, the focus of COPD treatment has slowly been shifting from acute, emergency care to care that emphasizes self-management and maintenance [8, 9]. Health coaching is a patient-centered and cost-effective model that has shown to be efficacious in treating other chronic diseases [10, 11]. Health coaches facilitate communication with providers and reinforce and individualize care plans. They work with patients for an extended period of time, and this continuity provides an opportunity for long-term behavior change, such as smoking cessation. Previous studies using health coaching techniques to address COPD have used registered nurses and respiratory therapists as health coaches with some success [12–14], but no studies known to the authors incorporate non-professionally licensed medical staff as health coaches.

This is the first randomized controlled trial using non-licensed health workers as health coaches to improve health outcomes and quality of life for patients with moderate to severe COPD. This manuscript reports the study protocol utilized in this randomized controlled trial. The results of this study will provide evidence about the efficacy and viability of a health coaching model by non-professionally licensed medical staff for the urban underserved and potentially offer a framework in which COPD care can be managed.

## Methods

### Study design

The Aides in Respiration (AIR) health coaching study is a multi-site, single-blinded randomized controlled trial. The study protocol was approved by the UCSF Human Research Protection Program (Approval#: 14-12872) and registered with clinicaltrials.gov (NCT02234284). An advisory board consisting of people living with COPD, healthcare practitioners from the study sites, and researchers in COPD care meet twice a year to guide the design and conduct of the study. One of the patient advisors acted as a key member of the study team, participating in intervention design, interviewing and hiring, and training and mentoring of the health coaches. An independent data safety monitoring board (DSMB) oversees the study and receives blinded reports every quarter to ensure there is no evidence of harm to the intervention group.

### Setting

This study is being conducted at seven urban county-operated primary care clinics that primarily serve a low-income, publically insured patient population. Two of these sites are large academic residency teaching practices based at the public hospital that is part of the county-owned system.

Pulmonary specialty care is available through the public hospital that is part of the health network and can be accessed via a referral system. Clinic sites have integrated behavioral health services. All sites have had prior exposure to health coaching for diabetes, hypertension, and/or complex care management programs.

### Participants

Patients are considered to meet clinical eligibility criteria if they have COPD, confirmed by post bronchodilator spirometry FEV1/FVC < 0.70 or review by a pulmonologist, that is moderate to severe. Moderate to severe COPD is defined as meeting at least one of the following criteria: at least one hospital admission in the last 12 months due to COPD-related diagnosis; at least two emergency department visits in the last 12 months due to COPD-related diagnosis; current prescription of an anti-cholinergic inhaler; current prescription of a combination long acting bronchodilator and inhaled corticosteroid inhaler; prescription of short term oral steroids (at least 40 mg for at least 4 days but less than 21 days) in the last 12 months; prescription of home oxygen therapy at any time; post-bronchodilator FEV1 % predicted < 80% at any time; outpatient O_2_ saturation < 88% at any time; or outpatient arterial blood gas (ABG/PPO_2_) < 55 mmHg at any time.

Non-clinical eligibility criteria are met if patients are at least 40 years of age, proficient in English or Spanish, contactable by telephone, and are currently receiving care and plan to continue to receive care at one of the seven study sites.

The health coaches have bachelor degrees from a four year college but are not licensed health care professionals. Both health coaches are fluent in English and Spanish.

### Identification and recruitment

Potentially eligible patients are identified from targeted diagnoses in billing records or hospital census data, as well as referrals from Chest Clinic and providers at study sites. Target diagnostic codes were: chronic bronchitis (491), emphysema (492), chronic airway obstruction, not elsewhere classified (496), bronchitis + tobacco use disorder (490 + 305.1), asthma + tobacco use disorder (493 + 305.1), and symptoms involving respiratory system and other chest symptoms + tobacco use disorder (786 + 305.1). Medical chart review is conducted for potentially eligible patients to determine whether they met clinical eligibility criteria.

Clinicians at each of the sites receive lists of their patients who are potentially eligible for the study and are asked to indicate patients who should be excluded from the study. Reasons for exclusion include severe or terminal health conditions, serious psychiatric or behavioral health issues that would prevent them from being able to work with a health coach, or other reasons the provider considered would prevent the patient from effectively participating in the study (e.g. patient not contactable by phone or transferring care).

Research assistants (RAs) contact all patients identified as potentially eligible by telephone using a recruitment script. RAs call each patient at least 5 times, with at least one attempt during each of the three timeframes: weekday mornings, weekday afternoons, and weekday evenings. Patients not reachable by phone are sent a letter signed by the patient’s clinic that explains the study and provides a phone number to call if the patient is interested in participation. Recruitment flyers in Spanish and English are posted in the clinics as an additional method of recruiting potentially eligible patients. In some cases, providers arrange to introduce a patient to an RA immediately following a medical visit in clinic.

Patients interested in the study are asked screening questions to confirm that the patient meets non-clinical criteria. If non-clinical criteria are met, then a time is set up to meet in person for enrollment.

If a patient does not have a record of post-bronchodilator spirometry documenting obstruction in their medical record, then post-bronchodilator spirometry is conducted to determine eligibility.

### Enrollment and randomization

RAs meet with eligible patients to explain the study and administer consent and HIPAA forms. The RA verbally administers a 60-min questionnaire. Data is collected on paper and then later entered into REDCap [15], a secure online web-based system for data collection. Clinical measures, such as spirometry and an exercise capacity test (the 6-min walk test), are collected if the patient does not present with contraindications. Pre-bronchodilator spirometry is conducted for patients with obstruction previously documented through post-bronchodilator spirometry.

**Fig. 1:**
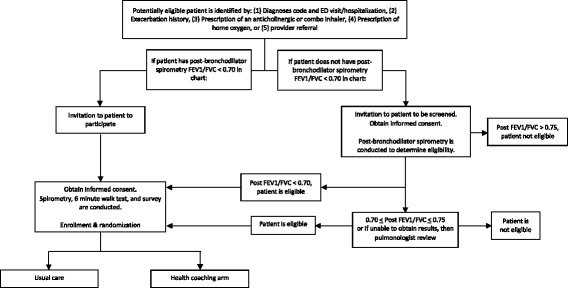
Study workflow from identification to enrollment

A random binary sequence, stratified by site, is used to order study arm assignment into sequentially-numbered envelopes. Once baseline measures are complete, the RA asks the patient to open a sealed envelope with a randomization card indicating whether the patient will be assigned to the usual care or health coaching arm. If a patient receives a health coach, the RA completes an intake form to give to a health coach, who then reaches out to the patient. Participants receive $10 for each measure (survey, exercise capacity test, and spirometry) at baseline, $10 for each survey at 3 and 6 months, and $20 for each measure completed at 9 months in acknowledgement of their participation in the study. This process from identification of potential patients to enrollment can be visualized in Fig. 1.

### Health coaching intervention

#### Health coach training and support

Health coaches receive over 100 h of training over 3 months using a COPD health coaching curriculum specific to the study. The curriculum is comprised of two primary domains: health coaching techniques and COPD-specific knowledge. The health coaching curriculum (available at http://cepc.ucsf.edu/content/health-coaching-curriculum) covers active listening and non-judgmental communication, harm reduction, navigating healthcare systems, gathering information on medication adherence, creating self-management goals, and closing the loop (checking for comprehension by asking patients to describe the key messages in their own words). COPD-specific training is delivered by two pulmonary specialists and covers the physiology of COPD, related comorbidities, Global Initiative for Chronic Obstructive Lung Disease (GOLD) guidelines, which are international guidelines for the management of COPD [16], prevention and management of exacerbations, and lifestyle management. Health coaches are given a comprehensive review of inhaled medications and medications related to smoking cessation. Particular emphasis is placed on how to observe and correct a patient’s inhaler technique and regimen. Training modules cover counseling methods for smoking cessation, as well as the importance of physical activity and pulmonary rehabilitation. Health coaches learn breathing techniques, such as pursed-lip and belly breathing, and the huff cough. In addition, health coaches meet with staff that have specialized knowledge in addressing social needs, mental illness, and environmental health for allergy/COPD management.

Upon completion of training, trainees must score at least 90% on three exams assessing content knowledge. In addition, trainees are required to demonstrate mastery of coaching skills through simulated role-plays and observations of health coaching sessions. Coaches have ongoing support from a pulmonary adult nurse practitioner (PNP) via weekly meetings at which they may present patient cases and request guidance with specific issues and may consult with the study investigators as needed.

#### Coaching overview

Once the health coaches complete the training and demonstrate mastery of the skills, they are assigned patients via the randomization of enrolled patients to the intervention arm. Each coach makes initial contact with patients randomized to the intervention arm at the time of enrollment to describe her role and to discuss areas of potential improvement for the patient. Each patient works with a health coach for nine months, with a total of fifty patients being assigned to each coach over the two-year duration of the study and a maximum caseload of thirty patients at any given time. Patient needs and preferences guide the frequency of contact, with a minimum suggested frequency of once every three weeks. Interactions between health coaches and patients are of three types: medical visits, individual visits, and phone calls.

#### Medical visits

Health coaches participate in medical visits between patients and their primary care and pulmonary clinicians. The health coach may meet with the patient immediately prior to the visit with the clinician to gather information about medication adherence and barriers to taking medications as prescribed, to identify agenda items of importance to the patient, and to ascertain breathing symptoms or recent exacerbations.

The health coach usually stays in the exam room during the medical visit. During the visit, the health coach may briefly supplement the patient’s summary with information learned during the pre-visit. In addition to taking notes on the visit, the health coach may act as an advocate: helping the patient to remember his or her questions and concerns; ensuring the patient’s vaccinations are up-to-date; sharing opportunities for praise, such as actions that the patient is taking to care for his or her health; or alerting the clinician to issues identified during the pre-visit, such as symptoms of an exacerbation.

After the medical visit, the health coach meets with the patient for a post-visit. The post-visit is used to “close the loop” with the patient about the care plan, ensuring that the patient can describe the care plan and recommendations in his or her own words. The health coach is responsible for facilitating navigation of other resources such as diagnostic imaging or referrals to specialists, making follow up appointments, or facilitating introductions to behaviorists or other clinic resources. In addition, if any behavior change is discussed in the visit, the health coach assists the patient in making action plans to, for example, incrementally increase physical activity, improve healthy eating, reduce stress, or improve medication adherence.

#### Individual meetings

In addition to medical visits, the health coach meets with the patient between visits by phone or in person at the primary care clinic, in the community, or in the patient’s home. The purpose of these visits or calls, generally lasting 15 to 90 min, is to set goals or address barriers to carrying out goals to assess patient knowledge, share information about target conditions, review inhaler use technique, and to assist with navigation of health and community resources.

Home visits are offered to patients, and are utilized most frequently by patients that have difficulties with public transportation or general mobility. Home visits are also used to identify COPD/asthma triggers within the home, acquire accurate knowledge of what medications a patient has in the home, including any duplicate or expired medications, identify barriers to medication adherence, and ensure patients on oxygen have the necessary equipment.

#### Phone calls

The majority of the patient-health coach interactions take place through phone calls. Each health coach has an encrypted cell phone used solely for the purpose of this study so that patients may directly contact their health coach as needed regarding any questions or concerns that a patient may have. Phone calls are most often utilized for appointment reminders, scheduling individual meetings, providing emotional support, following up on items discussed in a prior visit or phone call, and general wellness check in.

#### Optimizing COPD management

During one-on-one meetings with patients, health coaches also gather information from the patient regarding asthma symptoms, comorbidities, smoking history, and obstructive sleep apnea risk. COPD symptoms are assessed using either the COPD Assessment Test (CAT) or the mMRC dyspnea scale. The health coaches meet with a COPD PNP to present these findings and obtain treatment recommendations based on GOLD criteria. When appropriate, the PNP will communicate resulting recommendations with the PCP and/or the patient. Recommendations include changes to inhaler therapy, further diagnostic testing, and referrals to pulmonology, pulmonary rehabilitation, physical therapy, and other appropriate programs. The health coach will facilitate any recommended referrals and the implementation of any medication changes as necessary.

All interactions are documented in a database created for the study, including date, time, type, duration of contact, topics discussed, and any relevant notes.

### Usual care

Patients randomized to usual care continue to have visits with their primary care provider over the course of the 9-month period. They receive any resources their provider and their clinic offer as part of standard care, including but not limited to: access to COPD educators, respiratory therapists, COPD education classes, pulmonary rehabilitation, or smoking cessation classes.

### Measures

**Fig. 2:**
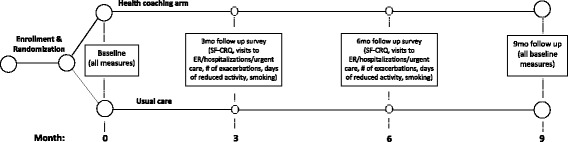
Participant timeline

Surveys at baseline, 3, 6, and 9 months capture demographics and patient-reported measures. Spirometric and exercise capacity data are collected at baseline and at 9 months. (Figure 2) The study research assistants use the VMAX Vyntus SPIRO with SentrySuite software to capture spirometric data. The Director of Community Spirometry reviews the spirometric data for quality and rated based on adherence to ATS criteria. A study pulmonologist then completes interpretations of the studies. The 6 min walk test (6MWT), which measures how far a patient can walk in 6 min, assesses exercise capacity following established protocol [17, 18]. Research assistants also review patient-presented medications and observe as patients demonstrate use of their inhalers. After completion of enrollment, chart review is conducted to identify prescribed medications, as well as hospitalizations and exacerbations for COPD in the year prior to enrollment.

Patient-reported measures include the Short Form Chronic Respiratory Disease Questionnaire [19], a chronic disease self-efficacy scale [20] adapted to COPD, COPD Assessment Test [21, 22], the short version of the Patient Assessment of Chronic Illness Care (PACIC) [23], the Patient Health Questionnaire (PHQ-8) [24], the Generalized Anxiety Disorder Scale [25], the Morisky Medication Adherence Scale [26, 27], the Trust in Physician Scale [28, 29], a single-item health literacy screener [30], and a modified version of an inhaler use checklist [31]. The timeline of these measures is shown in Table 1 below.

**Table 1:** Study measurements

	Study Period				
	Pre-Study	Enrollment	Follow up		
Timepoint	*-3 to -1 m*	*Baseline*	*3 m*	*6 m*	*9 m*
Activity:					
Eligibility screen	X				
Informed consent		X			
Randomization		X			
Measures:					
*6-min walk test*		X			X
*COPD Assessment Test (CAT)*		X			X
*COPD Knowledge*		X			X
*Days of reduced activity*		X	X	X	X
*Demographic questions*		X			
*Demonstration of inhaler technique (inhaler checklist)*		X			X
*Exacerbation History*		X	X	X	X
*Generalized anxiety disorder (GAD) scale*		X			X
*Morisky Medication adherence scale*		X			X
*Med concordance*		X			X
*Patient Assessment of Chronic Illness Care (PACIC)*		X			X
*Patient Health Questionnaire (PHQ-8)*		X			X
*Satisfaction with provider and clinic (SPC)*		X			X
*Self-efficacy(SE) scale*		X			X
*Short-Form Chronic Respiratory Disease Questionnaire (SF-CRQ)*		X	X	X	X
*Self-rated health (SRH)*		X			X
*Smoking*		X	X	X	X
*Spirometry*		X			X
*Trust in Physician (TIP) Scale*		X			X
*Visits to ED, hospitalizations, urgent care*		X	X	X	X

### Outcomes

The primary outcome is the mean dyspnea subscale score on the Short Form Chronic Respiratory Disease Questionnaire (SFCRQ). Secondary outcomes include the total SFCRQ score, self-efficacy for managing COPD, number of COPD exacerbations (an exacerbation is defined as a visit to an urgent care or emergency department for COPD, a hospitalization for COPD, or a prescription of an oral steroid and/or a course of antibiotics for worsening COPD symptoms), and exercise capacity as measured by the 6-min walk test. Additional measures include functional capacity measured by the COPD Assessment Test, quality of care measured by the Patient Assessment of Chronic Illness Care (PACIC), the number of reported sick days, smoking status, forced expiratory volume at 1 s (FEV1), knowledge of COPD, medication adherence, correct use of inhalers, and alignment of prescription medications to international GOLD guidelines.

### Quality assurance

The project manager conducts observations of each research assistant and health coach prior to the start of recruitment and periodically after to ensure compliance to study protocol.

Calibration of the Vyntus equipment is conducted daily using a 3-l syringe.

Survey data is entered into REDCap, which contains skip logic and specified ranges for entries. A logbook is also available in case further explanation is required to denote unusual circumstances.

### Sample size calculations

Sample size and power calculations were performed for the primary and secondary outcomes. Enrolling 190 patients and allowing for 20% attrition provided for power of 0.8 to detect a minimally important clinical difference (MCID) of 0.5 in SFCRQ dyspnea domain score and number of exacerbations and a power over .9 to detect a MCID of 0.5 for the total SFCRQ score and 50 m for the 6-min walk test using the standard threshold for a significant difference of .05 (2-sided).

### Statistical analyses

Intention-to-treat analyses will be performed for this study. Statistical analyses are run using Stata 13.0 (College Station, TX).

## Discussion

As the prevalence of COPD continues to rise, so does the cost of COPD to the healthcare system. Self-management has been shown to be an effective component of chronic care, yet limited resources have historically been available to provide self-management support. With COPD, the importance of specialty care adds yet another layer to the complexity to this care gap. Patients may face barriers to access to specialty care; even when specialty care is readily accessible, there are challenges to integrating specialty and primary care. Non-licensed health coaches, linked closely to pulmonary specialty providers, may serve as a financially feasible avenue through which this care gap could be remedied. This study protocol may provide evidence of the value of non-licensed health coach models to address COPD.

With the successful completion of the recruitment, this study is well equipped to measure the effectiveness of a non-professionally licensed health worker health coaching model in improving quality of life and clinical outcomes in patients with moderate to severe COPD in a safety net setting. This study will provide valuable insight into the efficacy of this model in addition to the strategies and challenges to implementation.

## Abbreviations

6MWT: 6 min walk test
ABG: Arterial blood gas
AIR: Aides In respiration
ATS: American Thoracic Society
CAT: COPD assessment test
COPD: Chronic obstructive pulmonary disease
FEV1: Forced expiratory volume at 1 s
FVC: Forced vital capacity
GOLD: The Global Initiative for Chronic Obstructive Lung Disease
MCID: Minimally important clinical difference
mMRC: Modified Medical Research Council
PACIC: Patient Assessment of Chronic Illness Care
PNP: Pulmonary adult nurse practitioner
RA: Research associate(s)
REDCap: Research electronic data capture
SF-CRQ: Short Form Chronic Respiratory Disease Questionnaire
