# Correction to: Health coaching to improve self-management and quality of life for low income patients with chronic obstructive pulmonary disease (COPD): protocol for a randomized controlled trial

**DOI:** 10.1186/s12890-019-0859-x

**Published:** 2019-05-21

**Authors:** Beatrice Huang, Rachel Willard-Grace, Denise De Vore, Jessica Wolf, Chris Chirinos, Stephanie Tsao, Danielle Hessler, George Su, David H. Thom

**Affiliations:** 1 Department of Family and Community Medicine, University of California San Francisco, San Francisco, CA USA; 2 San Francisco Department of Public Health, San Francisco, CA USA; 3 Department of Medicine: Pulmonology, Critical Care, Allergy and Sleep Medicine Program, University of California San Francisco, San Francisco, CA USA

**Correction to: BMC Pulm Med**

**https://doi.org/10.1186/s12890-017-0433-3**

In the original article [1], the authors reference their intention to use the ‘Morisky Medication Adherence Scale’. The scale is referenced in Table 1 and in the ‘Measures’ sub-section of the ‘Methods’ section.

This scale, however, was not used in the study.

The authors apologize for this error.
